# Assessment of the relationship between psychotic-like experiences and traumatic life events: a cross-sectional study

**DOI:** 10.1192/j.eurpsy.2024.1396

**Published:** 2024-08-27

**Authors:** S. Boudriga, M. Methni, S. Ben Nasr, A. Larnaout, U. Ouali, A. Aissa, J. Ventura

**Affiliations:** ^1^Razi, Manouba, Tunisia; ^2^Psychiatry A, Razi, Manouba, Tunisia; ^3^Department of Psychiatry and Biobehavioral Sciences, Semel Institute for Neuroscience and Human Behavior, Los Angeles, United States

## Abstract

**Introduction:**

Traumatic life events (TLEs) have been associated with the entire spectrum of psychosis outcomes, including risk and severity of psychotic disorders and psychotic-like experiences (PLEs). In a non-clinical setting, understanding the relationship could help improve prevention services.

**Objectives:**

The aim of this study is to establish the relationship between TLEs and PLEs.

**Methods:**

A cross-sectional study was conducted in a Tunisan business and engineering school from March 2022 to June 2022. Participants completed the Tunisian dialect version of the Prodromal Questionnaire-Brief (PQ-B), a validated self-report instrument designed to evaluate prodromal symptoms. TLEs such as physical, sexual, and emotional abuse, as well as neglect experiences, lived or witnessed have been assessed along with bullying experiences.

**Results:**

The final sample size consisted of 358 participants, with a median age of 22 ± 2.22 years, with a sex ratio (M/F) of 1.41. More than half of the participants (58.6%) reported having experienced TLEs (49% in the preceding 6 months) while 31% had experienced bullying or abuse in school (27.9% in the preceding 6 months). The mean total score of the PQ-B for the study population was 7.27 ± 4.387, 36.3% reached the threshold and were defined as PQ-B-positive subjects. Those with a lifetime history of major life events were more likely to screen positive on the total score PQ-B (p = 0.000), as were those with a lifetime history of bullying or abuse (p = 0.000).

**Conclusions:**

Understanding the factors that interact in the significant association between PLEs and TLEs may provide useful information for prevention programs and the improvement of mental health.

**Disclosure of Interest:**

None Declared

